# QuantiFERON conversion following tuberculin administration is common in HIV infection and relates to baseline response

**DOI:** 10.1186/s12879-016-1875-6

**Published:** 2016-10-07

**Authors:** Hanif Esmail, Friedrich Thienemann, Tolu Oni, Rene Goliath, Katalin A. Wilkinson, Robert J. Wilkinson

**Affiliations:** 1 Clinical Infectious Diseases Research Initiative, Institute of Infectious Diseases and Molecular Medicine, University of Cape Town, Cape Town, South Africa; 2 Department of Medicine, Imperial College London, London, UK; 3 Nuffield Division of Clinical and Laboratory Sciences, Radcliffe Department of Medicine, University of Oxford, Oxford, UK; 4 Department of Medicine, University of Cape Town, Cape Town, South Africa; 5 Division of Public Health Medicine, School of Public Health and Family Medicine, University of Cape Town, Cape Town, South Africa; 6 The Francis Crick Institute, London, UK

**Keywords:** Latent tuberculosis, HIV, QuantiFERON®-TB Gold in-tube, Diagnostics, Interferon Gamma Release Assay

## Abstract

**Background:**

HIV-1 infection impairs tuberculosis (TB) specific immune responses affecting the diagnosis of latent TB. We aimed to (1) determine the proportion of HIV-1-infected adults with a negative QuantiFERON®-TB Gold in-tube (QFT-GIT) and Tuberculin skin testing (TST) that convert to QFT-GIT positive following TST, and (2) evaluate the relationship between conversion and baseline QFT-GIT results.

**Methods:**

HIV-1 infected adults being screened for a TB vaccine study in South Africa underwent QFT-GIT followed by TST. As per protocol, QFT-GIT was repeated if randomization was delayed allowing for evaluation of TST boosting in a proportion of participants.

**Results:**

Of the 22 HIV-1 infected, TST and QFT-GIT negative adults (median CD4 477/mm^3^ IQR 439–621) who had QFT-GIT repeated after median 62 days (IQR 49–70), 40.9 % (95 % CI 18.6–63.2 %) converted. Converters had a significantly greater increase in the background subtracted TB antigen response (TBAg-Nil – all units IU/mL) following TST, 0.82 (IQR 0.39–1.28) vs 0.03 (IQR −0.05–0.06), *p* = 0.0001. Those who converted also had a significantly higher baseline TBAg-Nil 0.21(IQR 0.17–0.26) vs 0.02(IQR 0.01–0.07), *p* = 0.002. Converters did not differ with regard to CD4 count or ART status. ROC analysis showed a baseline cut off of 0.15 correctly classified 86.4 % of converters with 88.9 % sensitivity.

**Conclusions:**

Our findings support the possibility that there are 2 distinct groups in an HIV-1 infected population with negative QFT-GIT and TST; a true negative group and a group showing evidence of a weak Mtb specific immune response that boosts significantly following TST resulting in conversion of the test result that may represent false negatives. Further evaluation of whether a lower cut off may improve sensitivity of QFT-GIT in this population is warranted.

**Electronic supplementary material:**

The online version of this article (doi:10.1186/s12879-016-1875-6) contains supplementary material, which is available to authorized users.

## Background

The diagnosis of latent tuberculosis (TB) relies on the demonstration of an acquired cell-mediated immune response to *Mycobacterium tuberculosis* (Mtb) in an otherwise asymptomatic person, either by Tuberculin skin testing (TST) or Interferon gamma release assays (IGRA) (e.g., QuantiFERON®-TB Gold in-tube (QFT-GIT) and T-SPOT.*TB*) [1].

HIV-1 infection results in the depletion of Mtb specific CD4 cells [2] and impaired effector responses to Mtb antigens, which could result in a false negative test for latent TB. For TST, where diameter of skin induration is measured, a lower cut-off of 5 mm rather than 10 mm is therefore commonly used to improve sensitivity [3]. Alternatively a 2-stage testing approach, where the TST is repeated 1–4 weeks after an initial negative test, improves sensitivity. The initial TST is thought to boost a weak pre-existing antigen specific response, resulting in a positive test when repeated [4].

Unlike TST, IGRAs have adopted a single cut-off (QFT-GIT TBAg-Nil > 0.35 IU/mL, T-SPOT.*TB* > 5 spots) regardless of HIV-1 status. HIV-1 co-infection however has been shown to result in a reduced sensitivity of both IGRA platforms for tuberculosis [5, 6]. Boosting of interferon gamma release from circulating blood cells following TST has been observed within 3 days of administration [7]. This can result in conversion of IGRA from negative to positive, but occurs in less than 5 % of TST negative/IGRA negative cases in HIV-uninfected populations [8–11]. The impact of TST on IGRA conversion in immunocompromised persons such as those with HIV-1 infection has not been assessed.

In this study we evaluated the impact of TST on QFT-GIT conversion in HIV-1-infected persons that were initially TST and QFT-GIT negative and hence considered as not having evidence of latent infection. We then aimed to evaluate differences in baseline QFT-GIT response between those that converted and those that did not.

## Methods

### Study population

The study was conducted, in Khayelitsha, Cape Town, within the screening period of a TB vaccine trial, which enrolled participants between August 2011 and April 2013 [12]. The study adhered to International Conference on Harmonisation Good Clinical Practice guidelines, and was approved by the University of Cape Town’s Faculty of Health Sciences Human Research Ethics Committee. All participants provided written informed consent. Healthy HIV-1-infected adults with baseline CD4 counts above 350/mm^3^, if not receiving antiretroviral therapy (ART), or above 300/mm^3^ with suppressed HIV viral load, if receiving ART, were eligible for the vaccine trial. As part of the screening process all participants were assessed for active TB by symptom screen, chest radiograph (CXR) and sputum culture and for latent TB by QFT-GIT followed by TST. Those with no evidence of active or latent TB were eligible for randomization into the vaccine trial. However, if > 45 days had elapsed since screening initiation, the protocol determined that participants should be rescreened prior to randomization. This included repeating the QFT-GIT. In March 2012 there was a brief sponsor initiated suspension in recruitment to the vaccine trial. As a result when recruitment was reinitiated in April 2012 a number of screened participants breeched the 45-day rule and required rescreening. This pause in recruitment allowed assessment of boosting of the response following the initial TST presented here. The study was observational and did not have a control arm.

### Tuberculin skin testing and QFT-GIT

TST was administered as an intradermal injection of 2TU (0.1ML) of PPD-RT23 (Statens Serum Institut, Copenhagen, Denmark) into the volar aspect of the forearm and performed immediately after blood had been drawn for baseline QFT-GIT. Maximal transverse induration was measured at 48–72 h by clinical research nurses trained to do so. Induration < 5 mm was considered negative.

The QFT-GIT assay (Qiagen, Valencia, CA) was performed in accordance with manufacturer’s instructions. The following measures were implemented in order to minimise variation in assay results: (1) All samples were taken by research nurses trained and assessed to ensure consistent sampling methodology. (2) Following blood draw samples were kept in temperature-logged insulated transport boxes and samples were incubated within 4 h of blood draw. (3) Incubation time was standardized to 24 h. (4) QFT-GIT assay was performed by Good Laboratory Practice (GLP) trained research technicians in a Foundation of Innovative New Diagnostics (FIND) accredited laboratory with regular sponsor mandated Quality Assurance (QA) undertaken to ensure reproducibility of results.

### Statistical methods

Statistical analysis was conducted in Stata ver. 12.1 (StataCorp). Gaussian distribution of data was determined by the Shapiro-Wilk test. Non-parametric independent data was compared using Mann-Whitney *U* test and parametric data compared using *t*-test. Non-parametric paired data was compared by Wilcoxon signed-rank Proportions were compared by *χ* ^2^ test or Fisher’s exact test (if the contingency included a number ≤ 5). Receiver Operating Characteristic (ROC) analysis was performed as a non-parametric test.

## Results

Twenty-two HIV-1-infected adults screened for the vaccine trial with a negative TST and QFT-GIT required rescreening (due to brief suspension in recruitment) and therefore had a repeat QFT-GIT performed at a median of 62 days (IQR 49–70) after the TST. The TST was 0 mm in all participants. 90.9 % of participants were female and all were resident in Khayelitsha, a peri-urban township of Cape Town. Median age was 33 years (IQR 29–40). 16 participants (73 %) were on ART for a median of 2.0 years (IQR 1.5–3.5) and had a suppressed VL (either lower that detectable limit (LDL) or <40 copies/mL). Seven participants (31.8 %) had been treated for active TB a median of 3.7 years previously (IQR 2.0–4.2). Median VL for those not on ART was 17,309 copies/ml (IQR 10,563–34,735). Median CD4 count for the 22 participants was 477/mm^3^ (IQR 439–621). All participants had negative sputum culture and a CXR without evidence of active TB.

**Fig. 1:**
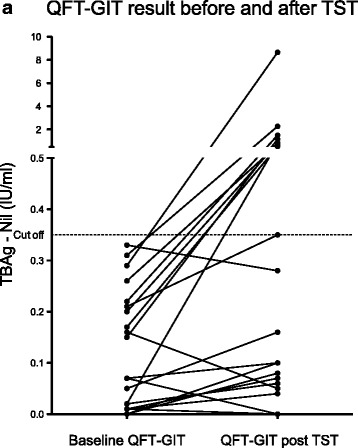
Change in QFT-GIT TBAg-Nil before and after TST. Graph showing TBAg-Nil level for participants at baseline and after boosting. Values for individual participants connected with line. Values <0 IU/mL not shown on graph

Nine of 22 participants (40.9 %, 95 % CI 18.6–63.2 %) demonstrated conversion from negative (<0.35 IU/ml) to positive QFT-GIT during the interval between tests. There was no significant difference in CD4 count, age, sex, proportion on ART, previous TB treatment or time between initial and repeat IGRA between those that converted and those that did not (Table 1). However, in those who converted, the median baseline TBAg-Nil of 0.21 IU/ml (IQR 0.17–0.26) was significantly higher compared to non-converters (0.02 IU/ml, IQR 0.01–0.07, *p* = 0.002) as was the change in TBAg-Nil post TST, 0.82 IU/ml (IQR 0.39–1.28) compared to 0.03 IU./ml (IQR −0.05–0.06, *p* = 0.0001), respectively. The repeat TBAg-Nil, post TST, was significantly higher in converters (0.84 IU/ml (IQR 0.65–1.50), *p* = 0.008) but not in non-converters 0.06 IU/ml (IQR 0.00–0.10), *p* = 0.86 (Fig. 1).

**Table 1:** Table showing differences in characteristics between converters and non-converters

Variable	Overall population *n* = 22	Converters *n* = 9	Non-converters *n* = 13	*p*-value
Age (years) – Med(IQR)	33(29–40)	32(29–40)	36 (30–39)	0.52
Sex - Female	90.9 %	100 %	84.6 %	0.49
CD4 count (/mm^3^) – Med(IQR)	477 (439 – 621)	466 (426–720)	477 (458–589)	0.87
On ART	73 %	66.6 %	76.9 %	0.66
Previous TB treatment	31.8 %	23.1 %	44.4 %	0.37
Time between tests (days) – Med(IQR)	62 (49–70)	50 (46–62)	69 (58–71)	0.11
Baseline TBAg-Nil (IU/ml) – Med (IQR)	0.07 (0.01–0.21)	0.21 (0.17–0.26)	0.02 (0.01–0.07)	0.002
Change in TBAg-Nil (IU/ml) – Med(IQR)	0.09 (−0.05–0.62)	0.82 (0.39–1.28)	0.03 (−0.05–0.06)	0.0001

Characteristics of 9 converters and 13 non-converters compared. Gaussian distribution of data determined by Shapiro-Wilk test. Non-parametric data compared using Mann-Whitney *U* test and parametric data compared using *t*-test. Proportions were compared by *χ* ^2^ test or Fisher’s exact test (if the contingency included a number ≤ 5)

*Med* Median, *IQR* Interquartile range, *ART* Antiretroviral therapy, *IU* international units

**Fig. 2:**
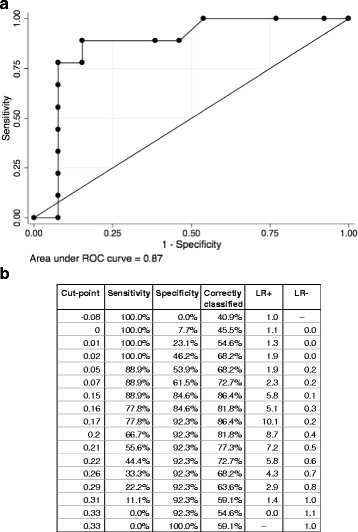
ROC analysis. **a**. Figure showing ROC analysis for baseline TBAg-Nil to distinguish converters and non-converters. **b** Table showing sensitivity, specificity, proportion correctly classified, positive likelihood ratio and negative likelihood ratio for each cut-off value. LR + = positive likelihood ratio, LR- = negative likelihood ratio

In order to determine if a specific cut-off could effectively identify converters, ROC analysis was performed on the baseline TBAg-Nil values. Area under the curve (AUC) of baseline TBAg-Nil to identify converters was 0.87 (95 % CI 0.69–1.00). TBAg-Nil cut-off values of 0.15 IU/ml and 0.17 IU/ml both correctly classified 86.4 % of converters, with 0.15 IU/ml cut-off having a higher sensitivity (88.9 % vs 77.8 %) and 0.17 IU/ml having a higher specificity (92.3 % vs 84.6 %) (Fig. 2).

## Discussion

We have shown that in an HIV-1-infected population living in high TB burden setting, over 40 % of those with an initially negative QFT-GIT (TBAg-Nil <0.35 IU/ml) and TST (<5 mm) convert to QFT-GIT positive (TBAg-Nil ≥0.35 IU/ml) within 7–10 weeks of a TST. We have also demonstrated that converters have a higher baseline QFT-GIT TBAg-Nil and that a baseline cut-off value of 0.15 IU/ml correctly classifies 86.4 % of converters with 88.9 % sensitivity. This rate of conversion is considerably higher than in previous studies conducted in a variety of settings with low HIV prevalence [8–11].

Although our study was conducted in a setting of high TB incidence it is unlikely that the 40 % conversion rate represents acquired infection over a 7–10 week period. The estimated force of infection in this setting is less than 8 % per year, therefore the expected rate of new infection over a 7–10 week period would be less than 1.5 % [13]. Furthermore if acquired infection was responsible for conversion, this would not be expected to relate to baseline QFN-GIT response.

Converters in our study had a significantly higher baseline TBAg-Nil than non-converters (0.21 vs 0.02). In addition to this, following TST, converters also had a significantly greater increase in TBAg-Nil with non-converters generally showing minimal change post TST (0.82 vs 0.03) demonstrating that TST did not just simply result in a uniform boosting in IFNγ production in all participants. The low baseline response and minimal boosting in non-converters in our study are similar to the responses in HIV-uninfected participants that are TST and QFT-GIT negative (<0.35 IU/mL) at baseline. Sauzullo et al. found 69 TST negative Italian healthcare workers to have QFT-GIT TBAg-Nil <0.03 IU/ml and <0.08 IU/ml before and 6 weeks after TST, respectively [8]. By contrast, previous studies have demonstrated that persons with positive QFT-GIT (>0.35 IU/mL) at baseline are more likely to show an increase in TBAg-Nil following TST than those with a negative QFT-GIT [14].

Taken together, our data supports the possibility that within this population of HIV-1-infected persons with negative TST and QFT-GIT there may be 2 distinct populations. A true negative group that have no evidence of immune sensitization by Mtb even following stimulation with mycobacterial peptides in the form of PPD and a false negative group that show initial evidence of weak antigen specific immune response to Mtb, as determined by IFNγ release in whole blood stimulated by ESAT-6, CFP-10, and TB7.7, that can be boosted by intra-dermal PPD injection. One explanation for this phenomenon is that pre-existing memory responses may be stimulated by PPD (which include antigens ESAT-6 and CFP-10) when TST is performed [15]. This may lead to proliferation of antigen specific effector cells which may then be present in greater numbers when peripheral blood is restimulated *ex vivo* when the repeat QFT-GIT is performed resulting in greater IFNγ release, and a positive QFT-GIT. Our data also suggest that a lower cut-off may improve sensitivity of QFT-GIT for the detection of latent TB in HIV-1-infected persons. Intuitively, having a lower cut-off in HIV-1-infected persons for an immune-diagnostic test is logical as the cells which contribute to a positive result are especially susceptible to HIV-1 induced depletion [2].

A limitation of the study was that we were unable to validate a lower cut-off for QFT-GIT in HIV-1-infected persons. It would also be important to ensure that any novel cut-off for HIV-1-infected persons was validated for a range of CD4 counts. Furthermore our study was only conducted using QFT-GIT, it would be of interest to determine if a similar phenomenon occurred using the TSPOT.*TB* assay. It was beyond the scope of the study to investigate whether participants that converted had any immunological differences in phenotype or function of antigen specific T cells in comparison to non-converters. In particular it would be of interest to investigate if converters had evidence of antigen specific central memory responses not present in non-converters as this would have supported the assertion that they represented distinct subgroups.

## Conclusion

We have demonstrated that in an HIV-1 infected population with negative QFT-GIT and TST (that would conventionally be characterised as not having latent TB infection and not considered for IPT) there are 2 distinct groups. One group shows evidence of a weak Mtb specific immune response that boosts significantly following TST resulting in conversion of the test result and may represent a false negative group that may benefit from IPT. We propose that a lower cut off, between 0.15 and 0.17, may improve sensitivity of QFT-GIT and warrants further validation.

## Additional file

Additional file 1: Table S1. Demographics and data for the 22 participants in study. (XLSX 63 kb)

## Abbreviations

ART: Antiretroviral therapy
CXR: Chest radiograph
FIND: Foundation of Innovative new diagnostics
GLP: Good laboratory practice
IGRA: Interferon gamma release assays
IQR: Interquartile range
IU: International units
Med: Median
Mtb: *Mycobacterium tuberculosis*
QA: Quality Assurance
ROC: Receiver operating characteristic
TB: Tuberculosis
TST: Tuberculin skin testing
